# Polysomnographic Features of Sleep Disturbances and REM Sleep Behavior Disorder in the Unilateral 6-OHDA Lesioned Hemiparkinsonian Rat

**DOI:** 10.1155/2014/852965

**Published:** 2014-12-25

**Authors:** Quynh Vo, Timothy P. Gilmour, Kala Venkiteswaran, Jidong Fang, Thyagarajan Subramanian

**Affiliations:** ^1^Department of Neurology, Penn State College of Medicine, Hershey, PA 17033, USA; ^2^Division of Engineering, John Brown University, Siloam Springs, AR 72761, USA; ^3^Department of Psychiatry, Penn State College of Medicine, Hershey, PA 17033, USA; ^4^Penn State Milton S. Hershey Medical Center, Mail Code H109, Room C2846, 500 University Drive, Hershey, PA 17033, USA

## Abstract

Sleep pattern disruption, specifically REM sleep behavior disorder (RBD), is a major nonmotor cause of disability in PD. Understanding the pathophysiology of these sleep pattern disturbances is critical to find effective treatments. 24-hour polysomnography (PSG), the gold standard for sleep studies, has never been used to test sleep dysfunction in the standard 6-OHDA lesioned hemiparkinsonian (HP) rat PD model. In this study, we recorded 24-hour PSG from normal and HP rats. Recordings were scored into wake, rapid eye movement (REM), and non-REM (NREM). We then examined EEG to identify REM periods and EMG to check muscle activity during REM. Normal rats showed higher wakefulness (70–80%) during the dark phase and lower wakefulness (20%) during the light phase. HP rats showed 30–50% sleep in both phases, less modulation and synchronization to the light schedule (*P* < 0.0001), and more long run lengths of wakefulness (*P* < 0.05). HP rats also had more REM epochs with muscle activity than control rats (*P* < 0.05). Our findings that the sleep architecture in the HP rat resembles that of PD patients demonstrate the value of this model in studying the pathophysiological basis of PD sleep disturbances and preclinical therapeutics for PD related sleep disorders including RBD.

## 1. Introduction

Nonmotor features of Parkinson's disease (PD) have become increasingly recognized. Disruption of sleep patterns is a major cause of daytime fatigue, daytime sleepiness, sleep related injuries, and associated disability in PD [1]. Sleep disturbances are seen in 80% of PD patients, and these symptoms may appear 10 years before the diagnosis of PD. Likewise, excessive daytime sleepiness and REM behavioral disorder (RBD) are risk factors for PD and there is strong connectivity between the extrapyramidal system and the sleep pathways [1]. RBD is characterized by excessive motor activity and absence of atonia during REM sleep [2]. There is emerging evidence that using premotor symptoms like RBD as a putative biomarker can allow for early symptom management and possible disease modification in PD. Furthermore, RBD affects quality of life in PD, causing serious injury while acting out vivid dreams, decreasing daytime wakefulness, and causing excessive fatigue. Treatments that target sleep signaling pathways have been shown to retard disease in animal models of PD, suggesting a beneficial potential for such interventions in humans at risk for PD [3].

A substantial amount of preclinical data exists in the rat HP model of PD that is not available in any other species. The largest amount of polysomnographic data and pathophysiology data for sleep disorders is also from the rat. For these reasons, we explored sleep dysfunction in the 6-hydroxydopamine (6-OHDA) unilateral medial forebrain-bundle (MFB) lesioned rat, the most commonly used and highly characterized rat model for PD. This model exhibits stable parkinsonian behaviors, drug induced dyskinesias, cortical and basal ganglia electropathophysiology, dopamine deficiency, and responsiveness to therapy. Despite the disadvantage that it is a relatively acute and static model of PD, its broad application, reproducibility, and easy availability make it an ideal model to study PD pathophysiology and experimental therapeutics. Surprisingly, 24-hour polysomnography had never been used in this commonly used preclinical model of PD to evaluate sleep disturbances. Our first aim was to fill this void by testing the hypothesis that HP rats would show sleep and wakefulness architecture that mimicked findings in untreated PD patients. Our second aim was to investigate and characterize the use of the unilateral 6-OHDA lesioned rats as an animal model to study RBD in PD by testing whether RBD-like sleep changes could be identified in 6-OHDA induced HP rats.

## 2. Methods

### 2.1. Rats and HP Induction with 6-OHDA

Twenty-four female Sprague-Dawley rats (220–250 g) were used in these experiments. Fifteen were used in the 24-hour principal component analysis (PCA) as we have previously described [4] and ten were used for RBD analysis. Experiments were carried out in accordance with the guidelines in the NIH Guide for the Care and Use of Laboratory Animals and approved by the PSUHMC IACUC. All surgery and procedures were done under deep general anesthesia and all efforts made to minimize suffering, to reduce the number of animals used, and to utilize alternatives to* in vivo* techniques, if available.

Ten rats in the HP group underwent stereotactic injection of 6-OHDA into the left nigrostriatal pathway using previously described protocols [4, 5]. All animals were pretreated prior to 6-OHDA injection with desipramine to avoid damage to norepinephrine (NE) pathways and to limit the lesion effect to the substantia nigra pars compacta (SNc) on one side. HP rats were challenged twice, 2 weeks apart, with apomorphine HCl (0.2 mg/kg, SC) to measure apomorphine-induced rotations (APIRs). Only rats with a mean of >245 rotations/35 minutes on 2 separate sessions were used in this study.

### 2.2. Polysomnographic (EEG/EMG) and Video Recordings

All rats were implanted with stainless steel skull screws over the frontal and parietal cortices for EEG recordings and with a pair of wire electrodes in the neck muscles for EMG recordings under general anesthesia as we have previously described [4, 5]. Rats were kept on a 12 : 12-hour light-dark cycle with free access to food and water. After 2 weeks of recovery and minimum 2-day adaption to the recording cable, 24-hour polysomnograms and videos were recorded from each rat. Each signal was amplified and digitized at 128 Hz and filtered offline in Matlab (Mathworks) and signals were segmented into 10-second epochs.

### 2.3. 24-Hour PCA Analysis

Each recording was scored offline by a trained human rater using the principal component analysis (PCA) sleep scoring method as we have described previously [4]. For the PCA scoring, seven features were computed from each epoch: absolute EEG power in the 1–4 Hz (delta), 5–9 Hz (theta), 10–20 Hz (low beta), and 30–40 Hz (high beta) bands and absolute EMG power in the 1–10 Hz band, theta-to-delta ratio, and beta-to-delta ratio. These seven features were collected into a feature matrix for each 24-hour recording. The three largest principal components were then computed and used to reduce the dimensionality of the feature matrix to three, and the projections were plotted in a three-dimensional scatterplot. The visual clusters were manually classified and the classification of the epochs was graphed. The secondary scatterplot with axes of the EMG, EEG high beta, and EEG delta was made available to the rater to aid in refining the scoring based on absolute EMG score as described previously [4].

Each sleep recording was 24 hours in length. The total time spent in each stage of sleep was computed, the percentage of time spent in sleep was calculated for each hour (zeitgeber time, aligned to “lights-on”), and the run lengths of each stage were calculated to examine the fragmentation level of the sleep. The percentage of time spent in sleep was compared with a two-way ANOVA using the factors of group (normal versus HP) and phase (dark versus light) or hourly values combined from combined rats as the main effect tests and also checking for an interaction. Individual post hoc *t*-tests were conducted if the hourly ANOVA was significant, using Tukey's Honest Significant Difference (HSD) correction. At the end of all sleep studies, animals were euthanized, and brains were cryosectioned and stained for tyrosine hydroxylase to verify unilateral >95% nigral dopaminergic cell loss.

### 2.4. EEG/EMG/Video Analysis

**Figure 1:**
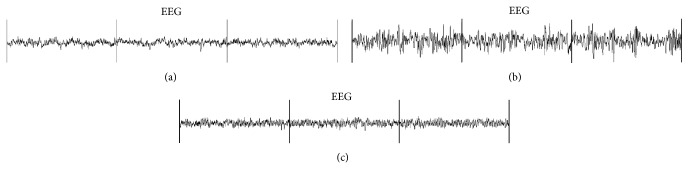
EEG patterns during different stages of sleep in three 10-second epochs of a normal rat: (a) wake, (b) NREM sleep, and (c) REM sleep.

**Figure 2:**
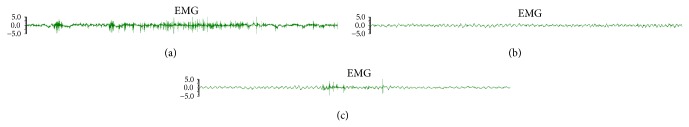
EMG recording during wake and REM sleep in 10-second epochs in an HP rat. (a) Muscle activity during wake. (b) Atonia during REM sleep. (c) Muscle activity during REM sleep.

For this analysis, we used 5 normal and 5 HP rats. In contrast to the 24-hour PCA analysis, only EEG recordings were used to determine the sleep stages of the rats in both groups (Figure 1). Each EEG recording was scored by two raters and REM sleep periods were correlated with time locked video behavior for confirmation of REM associated movements exhibited by the animals. EMG signals were analyzed directly using Spike2 (Figure 2). Two raters, blinded to each other's scores, were used to assess the REM sleep EMG recordings. The presence of muscle activity during REM sleep EMG recordings was defined as increased EMG level above baseline atonia for any length of time. Phasic muscle activity was not differentiated from tonic/nonphasic muscle activity. To assess for more complex behaviors during REM sleep, we examined video recordings of each animal for any observable movements during REM periods. These movements were readily distinguishable from arousal or wakefulness and from normal phasic REM movements.

We focused our analysis on the mean percentage of time spent in different sleep stages of the two groups. The percentage of time spent in each sleep stage and the percentage of REM epochs with muscle activity were compared between the groups using linear mixed-effects models in order to account for the repeated measurements. The percentage of REM epochs with movement on video was compared between the groups using a paired *t*-test. The level of probability for statistical significance was set at 0.05.

## 3. Results

**Figure 3:**
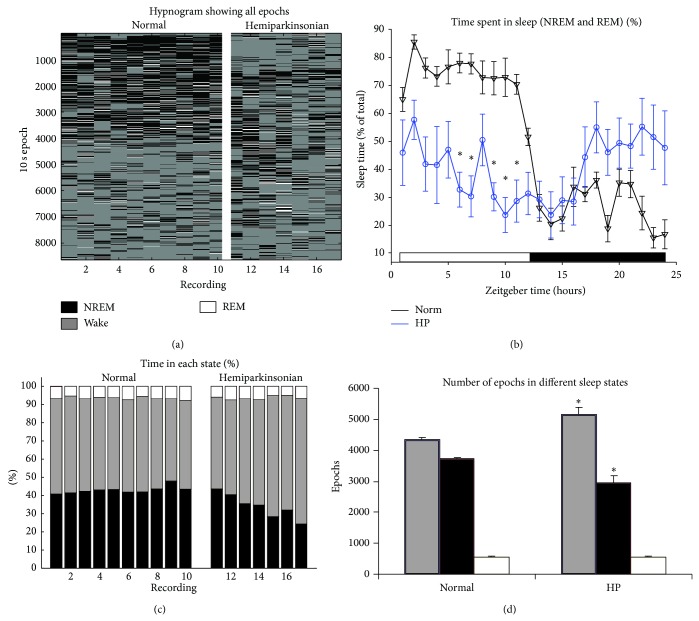
Overview of sleep structure in 24-hour recordings. (a) Plot of all sleep and wake 10-second epochs throughout the 24-hour recordings (numbered columns). Columns 1–10 show recordings from 10 normal rats (1 recording from each rat). Columns 11–17 show recordings from 5 HP rats (columns 11-12 and columns 13-14 show consecutive 24-hour periods from two HP rats, resp.). The top of the *y*-axis shows the beginning of the day when the lights were turned on. (b) Plot of hourly average sleep (combined NREM and REM) percentages across the entire 24 hours (error bars show SEM). (c) Percentages of epochs in NREM, wake, or REM states (colors and column labels are identical to (a)). (d) Number of epochs in different sleep states (^*^ *P* < 0.05, two-sided *t*-test between normal and HP rats; colors are identical to (a)).

From 24-hour PSG recordings, normal rats showed a standard pattern of 70%–80% time spent awake during the dark phase and 80% time spent asleep during the light phase (Figure 3). By contrast, the HP rats showed 30%–50% time spent asleep in both the dark and light phases. The two-way ANOVA showed a statistically significant difference in the percentage of sleep epochs, both between the dark and light phases (*P* < 0.0001) and between the normal and HP groups (*P* < 0.0001). Post hoc *t*-tests confirmed that there were significant hourly differences, especially in the latter half of the light phase. A statistically significant interaction between phase and group was also seen (*P* < 0.0001), confirming that the normal and HP rats responded differently to the light-dark cycle.

**Figure 4:**
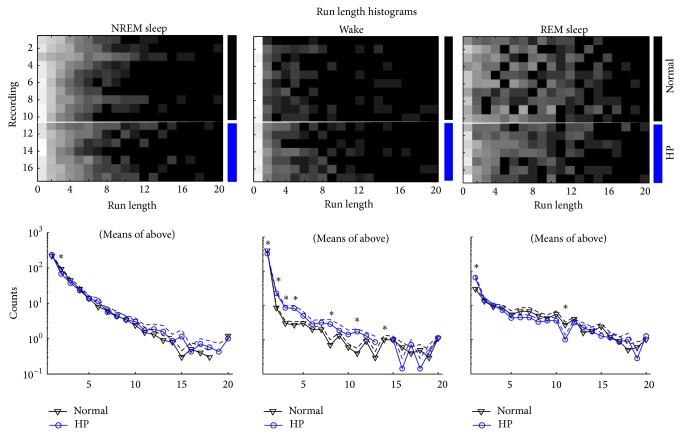
Run length comparison. Top: rows 1–10 show recordings from 10 normal rats (1 recording from each rat). Rows 11–17 show recordings from 5 HP rats (rows 11-12 and rows 13-14 show consecutive 24-hour periods from two HP rats, resp.). Columns show the run length (i.e., shorter run lengths on the left and longer run lengths on the right). Bottom: means (solid) and standard errors (dashed) of the top histograms showing normal (black triangles) and HP (blue circles). Missing data points on the log plots represent a mean of zero.

The HP rats also spent more total time in the wake state and less time in the NREM sleep state, compared to normal rats (Figure 3(d), *P* < 0.05). Additionally, the run length analysis showed several differences in the sleep structure (Figure 4). In particular, in the awake state, the HP rats had fewer single-length runs (i.e., 10-second epochs) and more runs of lengths 2, 3, 4, and 8 (i.e., 20-, 30-, 40-, and 80-second epochs, resp.) (there were no length-14 runs in the HP group).

Five normal rats and five 6-OHDA induced HP rats were used to assess the presence of muscle activity during REM sleep. One to four hours of recording was available for each rat, totaling five hours of data for the control group and fifteen hours for the HP group. Confirmation of identified REM periods with video recordings did not show any activity that indicated wakefulness. Thus, using EEG without EMG, we were able to determine the sleep stages for each animal. We first examined the distribution of time in each of the three wake-sleep stages as a percentage of total time recorded (Table 1). We were able to capture more periods of REM sleep with the HP group due to the longer recording; thus there was an insignificant disproportion between the HP group and the control group.

**Figure 5:**
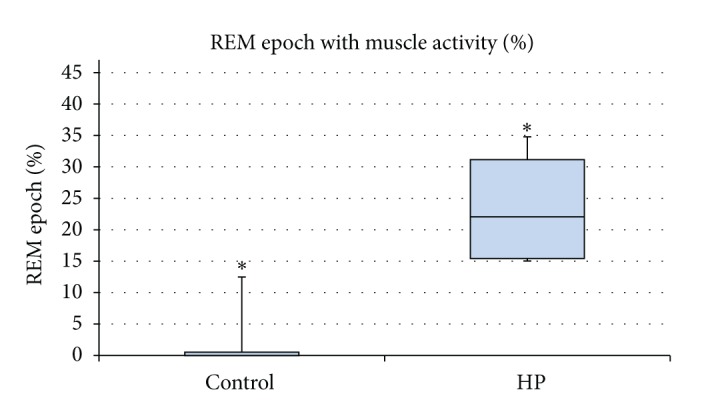
Percentage of REM epoch with muscle activity in control (*n* = 5) and HP (*n* = 5) groups. Comparison of mean difference indicates a significant increase in muscle activity during REM sleep in HP rats compared to control rats (*P* value 0.028).

**Figure 6:**
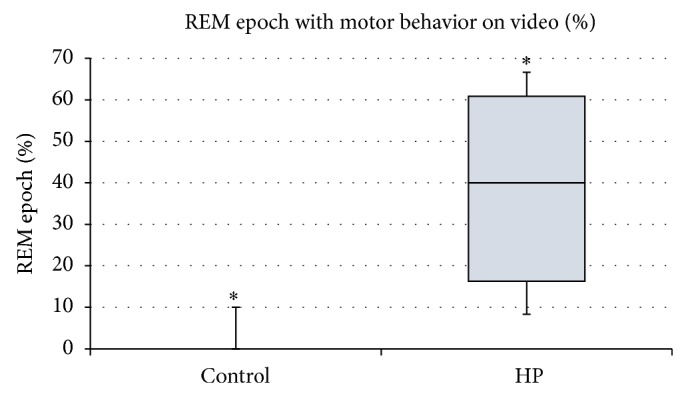
Percentage of REM epoch with motor behavior seen on video (*n* = 5) and HP (*n* = 5) groups. Motor behaviors included rhythmic head movements, sudden head movements, or sudden body movements that did not lead to arousal or wakefulness. Comparison of mean difference indicates a significant increase in movements observed on video during REM sleep in HP rats compared to control rats (*P* value 0.034).

We examined the distribution of muscle activity as a percentage of total time in REM sleep. Muscle activity during REM sleep was determined to be either absent (atonia) as expected for normal REM sleep or present indicating abnormal RBD-like behavior in each 10-second epoch. Muscle activity indicative of abnormal RBD-like behavior was present in all five HP rats. There was a significant difference in the occurrences of muscle activity during REM sleep in the HP group compared to the control group (Figure 5; *P* < 0.05). Further, on examination of video recordings during REM sleep, we observed motor behaviors that included rhythmic large head or body coordinated complex movements that lasted for significant length of time. These behaviors did not lead to subsequent arousal or wakefulness. These movements were much more robust and larger than minor muscle twitches reported with normal phasic REM sleep. There was a significant increase in the percentage of REM epochs with observable such motor activity in the HP group compared to the control group (Figure 6; *P* < 0.05). There was variability among the HP data. This is likely due to the small sample size with short recording periods.

Histological examination using cresyl violet (Nissl stain) and tyrosine hydroxylase (TH) immunohistochemistry as we have previously described showed that there was strictly unilateral SNc loss of TH-positive neurons that was ≥95% [5] and no evidence of histological lesions in the sublateral dorsal nucleus (SLD) or any areas that have been described in previously described models of RBD [1].

## 4. Discussion

This paper is the first 24-hour polysomnographic evaluation in the 6-OHDA lesioned HP rat, a standardized, commonly used animal model of PD. The sleep architecture changes in the HP rat closely resemble the sleep architecture described in PD patients demonstrating the applicability of the unilateral HP rat model for pathophysiological studies of sleep dysfunction in PD. Our study is novel in that it identified sleep behavioral abnormalities that closely resemble RBD (RBD-like) in the unilateral 6-OHDA induced HP rats as evidenced by increased muscle activity and visible movements during REM sleep epochs. These differences were significantly different in HP rats when compared to control rats.

The origin of RBD in PD is currently unknown. In the rat model, the sublateral dorsal (SLD) nucleus, equivalent to the locus subcoeruleus in humans, is the major structure responsible for REM sleep. Lesion studies of the SLD have resulted in REM sleep without atonia [6], leading Boeve and colleagues to hypothesize the SLD to be the final common pathway inducing muscle atonia during REM sleep [7]. Similarly, PD patients with RBD showed structural pathological changes in the locus coeruleus on autopsy [8, 9]. Thus, the involvement of the locus coeruleus in PD may explain the origin of RBD in PD. However, RBD is most often seen much earlier in PD when the disease is in its preclinical stage or in stage I (unilateral parkinsonism). At this stage of the disease, there is very little evidence of damage or pathology to the locus coeruleus in PD. Moreover, the role of nigrostriatal degeneration, the dominant pathology in PD, on RBD associated with PD is not well understood.

Animal models of PD have been proposed for the study of sleep disturbances in PD [10, 11]. Yi and colleagues infused rotenone subcutaneously for one month in rats and showed with EEG and EMG recordings that these rats exhibited insomnia during their normal daytime sleep period and excessive drowsiness during their normal nighttime awake period [12]. However, in these experiments, the animal developed exceedingly mild locomotor dysfunction (<5% reduction in locomotor activity) and this effect was bilateral and symmetric. This is not reflective of onset of disease in PD, which clinically starts with marked unilaterality, a diagnostic hallmark of the disease (definition of stage I disease). McDowell and colleagues proposed a model utilizing cycad seed flower to generate a sleep dysfunction in the rat that mimicked PD associated sleep disturbances [13]. However, these animals never developed any motor findings of PD. Barraud and colleagues reported circadian disruptions in the 2 MPTP-lesioned severely parkinsonian monkeys and sleep dysfunction that closely resembled the sleep disruptions seen in PD patients [14]. However, it is still unclear if these changes in REM sleep are related to RBD seen in PD as the severity of the disease state in these animals could easily have caused the excessive fatigue and sleep disturbances. Thus, this animal model does not appear to mimic RBD that is seen in early clinical PD.

6-OHDA use in animal models allows specific targeting of lesions to mimic parkinsonian motor symptoms. Gravotta and colleagues, while targeting widespread dopaminergic (DA) fibers with injections of 6-OHDA into the third ventricle, indicated that DA plays a prominent role in regulation of circadian activity [15]. Additionally, the study assessed sleep/wakefulness using video motion analysis and wheel-running activity has been tested in the HP rat previously. However, it did not use PSG to evaluate sleep architecture or to determine sleep onset latency and sleep run lengths during periods of wakefulness as we report. Ben and Bruguerolle found a circadian rhythm disruption in rats following bilateral striatal 6-OHDA injection that persisted at 4 weeks using a heart rate measure [16]. Monti and colleagues reported an increase in sleep in their 6-OHDA ventricle-injection rat model. However, they only analyzed 6-hour blocks, not the full 24-hour periods that we analyzed [17]. Disturbances in the sleep-wake architecture characterized by increased wakefulness and nocturnal limb movement after bilateral striatal 6-OHDA lesions have been reported [18]. These animal models (systemic MPTP injections, ventricular 6-OHDA, and the bilateral 6-OHDA) cause severe heterogeneous expression of parkinsonism, associated with debilitating disability and significant morbidity. This severe morbidity could have contributed to the sleep deficits in these animal models [19].

Our study demonstrated changes in the sleep-wake architecture with visible movements suggestive of RBD with unilateral 6-OHDA lesioning of the nigrostriatal pathway. Histology showed exclusive deficits of dopamine neurons in the nigrostriatal pathway, with no abnormality in other neurotransmitter pathways. This suggests that unilateral nigrostriatal dopamine depletion is sufficient to produce PD related sleep abnormalities and RBD-like behavior. Moreover, our study also suggests that bilateral lesioning of the nigrostriatal pathways is not required to cause sleep abnormalities. Taken together, our study strengthens the notion that pathophysiology of sleep disturbances in PD is mediated via the nigrostriatal pathway and dopamine replacement, particularly during sleep, may be a meaningful way to address PD associated sleep dysfunction.

Further, the unilateral 6-OHDA lesioned HP rat model has very low morbidity and has been extensively characterized for its easily reproducible parkinsonian deficits [20]. This model can be generated in large numbers easily making them very suitable for translational research studies. These animals had full ability for self-care, feed, and drink independently and demonstrate normal exploratory behavior. Therefore, this model facilitates the detection of PD related sleep dysfunction without the presence of severe motor impairment, which is a confounder for sleep studies in PD models with more severe parkinsonian morbidity.

Our results may also have correlated to electrophysiological recordings in anesthetized HP rats. Recent studies in HP rats have shown that abnormal electrophysiological burst patterns, neuronal firing entropy, and oscillations in the subthalamic nucleus and in the substantia nigra reticulata in slow wave sleep state improve towards normalcy with dopaminergic therapy. Such benefits from dopaminergic therapy were not noticed in the global activation state [5]. The sleep architecture dysfunction demonstrating decrease in NREM sleep in the HP rat may represent the effects of unmitigated dopamine deficiency. If cortical slow wave activity during urethane anesthesia is the equivalent of NREM stage of normal sleep as many hold to be true, then NREM abnormalities we report in this paper may be responsive to dopaminergic therapy. Future studies that evaluate the effects of dopaminergic and nondopaminergic therapies in the 6-OHDA lesioned HP rat using PSG and depth electrode recordings may provide significant clues on the pathophysiology of sleep dysfunction in PD, a major cause of disability in patients.

There are several drawbacks to our study which need to be addressed in future work. First, eye movements were not recorded in these rats using EOG, and cardiac and respiratory monitoring was also not available. These parameters would increase the accuracy of REM sleep identification and help to differentiate tonic and phasic REM sleep in the animals. Unlike the nonhuman primate that exhibits a posterior dominant rhythm in wakefulness and other sleep hallmarks similar to those of humans, the rat does not have such EEG findings. EMG was obtained only from the proximal trunk and neck muscles in our study. The technical limitations of our study set the stage for future studies to investigate this relationship in more detail, using advanced miniaturization and implantable telemetry devices, so that other physiological parameters may simultaneously be recorded for analysis.

Second, in the current study, we focused on a small portion of the recorded data. Studies that include a larger sample size and multiple 24-hour periods of comprehensive sleep recordings may be useful to reduce the variability with this model as reported by others [11, 21] and in our study for muscle activity and video ratings of REM behavior (Figures 5 and 6).

Third, although we found a significant difference in the sleep architecture and RBD-like behavior in HP rats, we did not test the responsiveness of these abnormalities to dopaminergic therapy. Further work should examine the response of sleep abnormalities and RBD-like behavior in this model to currently available treatments for PD and RBD.

In summary, our study provides evidence that the commonly used 6-OHDA lesioned HP rat model exhibits sleep abnormalities that are common in human PD, including RBD-like behavior. The impact of this finding is profound, as new studies in this model can tap into the extensive repertoire of preexisting PD related and sleep related published data in the rat and potentially provide a tool to study the pathophysiology and preclinical experimental therapeutics for sleep abnormalities and RBD in PD, an unmet need in the field [3].

## Figures and Tables

**Table 1:** Comparison of mean differences of percentage of time spent in wake-sleep stages (wake, NREM, and REM), percentage of REM epochs with muscle activity, and percentage of REM epochs with observable motor activity between control and hemiparkinsonian (HD) groups.

	Group 1	Group 2	Mean difference	95% confidence interval	*P* value
			(Group 1-Group 2)		
% time in wake	HP	Control	−30.69	−46.28, −15.09	0.001
% time in NREM	HP	Control	20.82	7.57, 34.08	0.005
% time in REM	HP	Control	9.86	6.54, 13.18	<0.001
% REM epoch with muscle activity	HP	Control	20.54	2.62, 38.45	0.028
% REM epoch with observable motor behaviors	HP	Control	36.43	6.46, 66.40	0.034
